# The Critical Role of Acyl Hydrocarbon Receptor on the Combined Benefits of Postbiotic Propionate on Active Vitamin D3-Orchestrated Innate Immunity in *Salmonella* Colitis

**DOI:** 10.3390/biomedicines11010195

**Published:** 2023-01-12

**Authors:** Fu-Chen Huang, Shun-Chen Huang

**Affiliations:** 1 Department of Pediatrics, Kaohsiung Chang Gung Memorial Hospital and Chang Gung University College of Medicine, Kaohsiung 833, Taiwan; 2 Department of Anatomic Pathology, Kaohsiung Chang Gung Memorial Hospital and Chang Gung University College of Medicine, Kaohsiung 833, Taiwan

**Keywords:** acyl hydrocarbon receptor, postbiotics, active vitamin D3, innate immunity, *Salmonella* colitis

## Abstract

Our recent study observed the combined beneficial effects of postbiotic butyrate on active vitamin D3-orchestrated innate immunity to *Salmonella* Colitis. There is increasing interest in the role of acyl hydrocarbon receptor (AhR) on colitis and innate immunity. Therefore, we investigated the involvement of AhR in the effects. *Salmonella* colitis model is conducted with 6–8 w/o male C57BL/6 mice: Streptomycin (20 mg/mouse p.o.)-pretreated C57BL/6 mice were mock infected with sterile PBS or infected orally with 1 × 10^8^ CFU of an *S. typhimurium* wild-type strain SL1344 for 48 h. Before and after the colitis induction, mice were oral gavage with active vitamin D3 0.2 μg/25 g mice (VD3) and/or postbiotics propionate (PP), in the absence of the presence of intraperitoneal injection of AhR inhibitor for 4 and 7 days, respectively. We observed AhR inhibitor counteracted the synergistic effects of PP and VD3 on reducing the severity of *Salmonella* colitis and body weight loss in C57BL/6 mice, reducing the cecal inflammatory but enhancing antimicrobial peptide mRNAs expression, and reducing the bacterial translocation in liver/spleen, compared to single treatment. It suggests the involvement of AhR on the synergistic effects of postbiotics PP and VD3 on the antibacterial and anti-inflammatory responses in *Salmonella* colitis and the potential biological treatment of *Salmonella* colitis.

## 1. Introduction

*Salmonella* spp. are important Gram-negative food-borne pathogens of humans and animals. If treatment is delayed or inadequate, severe systemic infections may lead to high-mortality complications including meningitis, osteomyelitis, sepsis, and toxic megacolon. Some *Salmonella* strains are becoming increasingly resistant to antibiotics, which may be associated with an increased risk of hospitalization, development of a bloodstream infection, or treatment failure. The trends of increasing incidence of food-borne human infections caused by multi-drug-resistant strains of *S. typhimurium* were noted globally [1] including in Taiwan [2]. Excess mortality was associated with antimicrobial drug-resistant *S. typhimurium* [3].

Medical nutrition therapy (MNT) is an evidence-based, individualized nutrition process meant to help treat certain medical conditions. MNT is based on decades of medical research on the relationship between diet, nutrition, and health outcomes. As chronic diseases become more prevalent, with prolonged and changing lifespans, MNT is a key scientific platform to improve clinical symptoms and reduce inflammation, leading to the induction and/or maintenance of disease remission, and finally, promoting health and preventing diseases. Inventor Thomas Edison said that “the doctor of the future will give no medicine but will interest his patients in the care of the human frame, in diet and in the cause and prevention of disease”.

Accumulating evidence indicates that nutrition can modulate the immune system through metabolites, either produced by host digestion or by microbiota metabolism. Food represents not only a source of nutrients for the maintenance of essential biological functions but also contains dietary components that regulate immune cell populations. Aryl hydrocarbon receptor (AhR) is a ligand-activated transcription factor that integrates environmental, dietary, microbial, and metabolic cues to control complex transcriptional programs. An important source of nutritional AhR ligands is microbiota metabolism, that is, postbiotics. Examples of postbiotics include short-chain fatty acids (SCFAs), such as acetate, butyrate, and propionate (PP). SCFAs, which originate from the fermentation of dietary fibers by the microbiota, can activate AhR signaling.

The role of AhR in colitis and bacterial infection is increasingly brought on stage. Many fruits and vegetables contain naturally acquired compounds that might be AhR ligands, such as quercetin in apples, and resveratrol in red wine. This makes AhR an important environmental sensor to initiate transcriptional regulation in response to dietary factors. Treatment of 6-formylindolo [3,2-b]carbazole (FICZ) (an AhR ligand) significantly decreased the severity of chemical-induced colitis in mice, which was characterized by down-regulation of pro-inflammatory cytokines [4,5]. In contrast, treatment of mice with an AhR antagonist enhanced the severity of inflammation in trinitrobenzene sulfonic acid-induced colitis. Indeed, AhR deficiency in mice results in higher susceptibility to *Citrobacter rodentium* [6,7,8], which is a natural mouse pathogen widely used to mimic enteropathogenic and enterohemorrhagic *Escherichia coli* (*E. coli*) infections in humans [9].

Our recent study [10] observed the combined effects of butyrate and active 1,25-dihydroxyvitamin D3 (VD3) on antimicrobial peptides (AMPs) and anti-inflammatory responses in *Salmonella* colitis [10]. However, the generalization of the combined benefits of postbiotics and VD3 on *Salmonella* colitis and invasiveness, and the role of AhR on the benefits are not yet reported. Therefore, we investigate the role of AhR on the synergistic effects of postbiotics and VD3 on the severity of *Salmonella* colitis and invasiveness in mice.

## 2. Materials and Methods

### 2.1. Bacterial Strains

The naturally streptomycin-resistant wild-type strain *S. enterica* serovar Typhimurium SL1344 (*S*. Tm) was grown for 2 h at 37 °C in Lysogeny broth supplemented with 50 μg/mL streptomycin, diluted 1:100 in fresh broth, and sub-cultured for 16 h at 37 °C under mild aeration. Then, bacteria were washed twice in PBS and suspended in PBS to 10^9^ CFU/mL.

### 2.2. Reagents

The propionate was obtained from Sigma (St. Louis, MO, USA). Active 1,25-dihydroxyvitamin D3 (VD3) (Biomol Research Laboratories, Plymouth, PA, USA) was stored as a stock solution in pure ethanol at 22 °C in the dark. Standard laboratory reagents were from Sigma (St. Louis, MO, USA) or Fisher Scientific (Pittsburgh, PA, USA).

### 2.3. Postbiotics Preparation

Sodium propionate powder was purchased from Sigma-Aldrich (Merck KGaA, Darmstadt, Germany) and stored at room temperature. The dose used for animal experiments was 1% or 5% propionate. The propionate powder was weighed and dissolved in sterile ddH_2_O. After filtering the solution with a 0.45 μm filter, the stock was dispensed in a vial and stored at −20 °C for further use.

### 2.4. AhR Inhibitor Solution Preparation

The aryl hydrocarbon receptor inhibitor (CH-223191) powder was purchased from Sigma-Aldrich (Merck KGaA, Darmstadt, Germany) and stored at −20 °C. The powder of AhR inhibitor was weighed, dissolved in DMSO as the stock solution, and stored at −20 °C. For preparing the injection solution, dilute the stock in 1× PBS buffer. DMSO in 1× PBS buffer was used as vehicle.

### 2.5. Animal Experiments

All mice were generously provided by the National Laboratory Animal Center. Forty-two 6–8-week-old female C57BL/6 mice under specific pathogen-free conditions were fed in an SPF room at Kaohsiung Chang Gung Memorial Hospital animal center. Animal experiments were approved by the Kaohsiung Chang Gung Memorial Hospital Institutional Animal Care and Use Committee according to the legal requirements. For the experiments, the mice were housed in individually ventilated cages and transferred to a negative-pressure room. Mice were divided into these groups: Control (open control), SL (*S*.Tm infected), VD (VD3 and *S*.Tm infected), PP1 or PP5 (1% or 5% propionate and *S*.Tm infected), VD + PP1 (combination of VD3 and 1%propionate plus *S*.Tm infected), VD + PP5 (combination of VD3 and 5% propionate plus *S*.Tm infected), VD + PP5 + AHRi (combination of VD3 and 5% propionate plus *S*.Tm infected and AhR inhibitor).

Before the colitis induction, mice were oral gavage given VD3 0.2 μg/25 g mice (VD) or postbiotics PP or a combination of both in the absence or presence of intraperitoneal injection of AhR inhibitor (AHRi) for 4 days. Other groups were given 100 μL sterile water (open control) or 100 μL PBS (SL group) and intraperitoneal injection of DMSO solution.

Water and food were withdrawn 3 h before oral gavage treatment with 20 mg streptomycin (100 μL sterile water for open control). Then, animals were supplied with water and food at will. Twenty-four hours after streptomycin treatment, food and water were withdrawn again for 3 h and infected with *S. enterica* serovar Typhimurium SL1344 10^8^ CFU (suspend in 100 μL PBS) or treated with sterile water (100 μL sterile water for open control). Food and water were supplied at will again. After infection at 48 h, the mice were again treated with VD3 0.2 μg/25 g mice (VD) and/or PP and/or intraperitoneal injection of AhR inhibitor for 7 days. Other groups were given 100 μL sterile water (open control) or 100 μL PBS (SL group) and intraperitoneal injection of DMSO solution. On day 14, submandibular bleeding was collected using lancets. Then, mice were sacrificed by CO_2_ asphyxiation and the tissue samples from the intestinal tracts, spleens, mesenteric lymph nodes, and livers were collected for analysis (Supplementary Figure S1).

Animal body weight and diarrhea situation scores were recorded during the experimental process. The diarrhea situation was scored as follows: 5 = mice live energetic; 4 = mice occurred diarrhea and pasty stools; 3 = mice occurred loose stools and reduced mobility; 2 = mice live weak and occurred abnormal behavior; 1 = mice lost their lives. We also calculated the spleen index as an assessment of immunity.

### 2.6. Histological Colitis Scoring

Postmortem, the entire colon was removed, and the colon length and weight were measured. Segments of cecum were harvested and fixed in 10% formalin (pH 7.4) and embedded in paraffin according to a standard protocol. Sections (5 μm) were stained with hematoxylin and eosin (H&E). Blinded histological scores were performed by a validated scoring system by a trained pathologist. H&E sections were scored based on a study reported by Barthel et al. [11]. The combined pathological score for each tissue sample was determined as the sum of the following scores; submucosal edema (score, 0–3), polymorphonuclear granulocytes in the lamina propria (score, 0–4), number of goblet cells (score, 0–3), and epithelial integrity (score, 0–3) [11].

### 2.7. Immunohistochemistry (IHC) Staining Procedures

Paraffin sections of paraffin-embedded tissue samples from the cecum of each animal were mounted on glass slides. After deparaffinized and rehydrated, the slides were microwaved in a retrieval buffer for the purpose of antigen retrieval. The slides were then blocked in 10% normal serum with 1% BSA in TBS for 2 h at room temperature. After draining for a few seconds, the slides were then incubated with the primary antibody at 4 °C overnight. The slides were rinsed with TBS buffer a few times. Subsequently, slides were incubated for 1 h at room temperature with secondary antibody (HRP-conjugated antibody). The slides were rinsed and incubated with the chromogen 3,3′-diaminobenzidine to visualize the target protein and counterstained with hematoxylin. The slides were dehydrated, cleared, and mounted for further analysis.

### 2.8. Immunohistochemistry Staining Analysis

An automated whole-slide scanning device (3DHISTECH, Sysmex, Switzerland) and software (Pannoramic viewer, Sysmex, Switzerland) were implemented in our workflow. The scanned images were analyzed using the free software ImageJ Fiji. Semi-quantitative IHC (Bio-protocol, Wei Yue, 2019) is a powerful method for investigating protein expression within tissues. By using the software ImageJ Fiji, we conducted deconvolution and downstream analysis. The area of the scanned images that was interesting was circled and measured by a trained pathologist. Ten interested regions were chosen from every slide image and at least three experiments were performed to collect the values of images for further statistical analysis.

### 2.9. Quantitative Real-Time PCR Analysis of Cecum or Cultured Cells RNA

Samples of the cecum were obtained, immediately snap-frozen in liquid nitrogen, and stored at −80 °C. Total RNA was extracted from the cecum tissue using TRI Reagent (Ambio #15596018) and the Directzol RNA Miniprep Kit, according to the manufacturer’s instructions. The RNA was reverse-transcribed into cDNA as previously described in detail [10,12]. Then, cDNA samples were subjected to quantitative real-time PCR using ABI 7500 Real-Time PCR System (Applied Biosystem) and FAST SYBR GREEN MASTER MIX according to the manufacturer’s directions. The primers for the mouse genes of interest and reaction protocol were set as in previous reports [10,12], except for mouse aryl hydrocarbon receptor (AhR), forward, 5′-ACATCACCTATGCCAGCCG-3′, reverse, GACTTAATTCCTTCAGCGGGGA-3′. The relative number of transcripts was normalized to the amount of β-actin transcript by subtracting the mean Ct value of the latter from the mean Ct value of the former for each experimental condition. The difference between the normalized Ct values of the infected or treated samples and the control ones is a measure of the change in mRNA expression. Many aspects of the MIQE guidelines were taken into consideration for the methods and analysis [13].

### 2.10. Statistical Analysis

All the above experiments were performed in triplicate with similar results. We made use of GraphPad Prism 8 software (GraphPad Software, San Diego, CA, USA) to perform the statistical analysis. By using the software GraphPad Prism, each data is tested to see if it fulfills the normal distribution using normality and lognormality tests. If the data were parametric, RM one-way ANOVA with the Geisser–Greenhouse correction was used to analyze the data. If they were nonparametric, then the Friedman test was used. A *p*-value of <0.05 was considered statistically significant.

## 3. Results

### 3.1. The Involvement of AhR in the Synergistic Effects of Postbiotic PP on VD3-Mediated Reduced Severity of Salmonella Colitis

**Figure 1:**
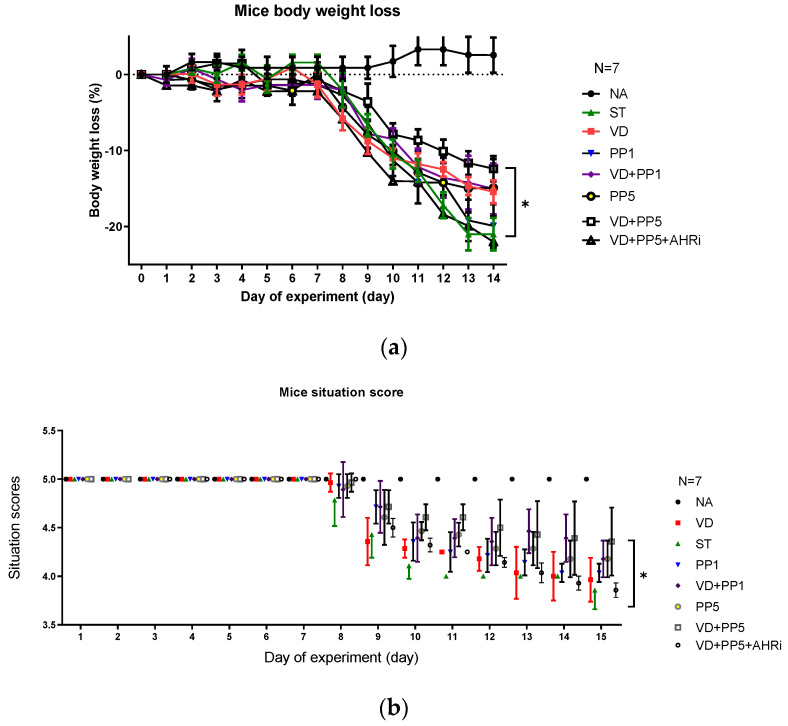

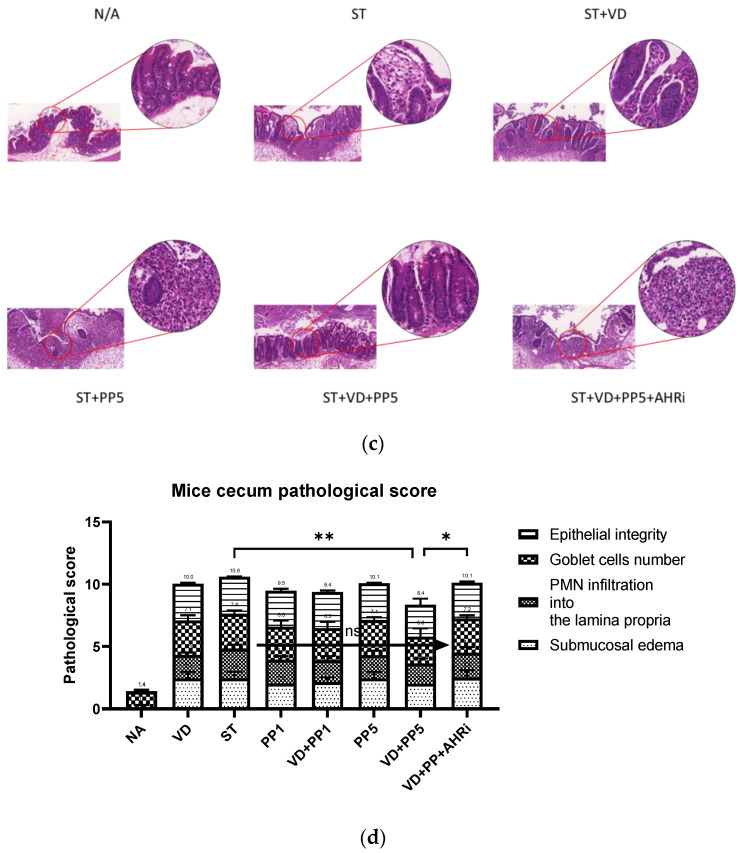
The involvement of AhR in the synergistic effects of PP on VD3-mediated attenuating the severity of *Salmonella* colitis in mice. Six–eight-week-old female C57BL/6 mice (Charles River, Wilmington, MA, USA) were bred and housed under specific pathogen-free conditions in the animal facility of the Center for Cellular and Biomolecular Research, Kaohsiung, Taiwan. Mice were treated or infected as in Materials and Methods and divided into the following groups: Control (NA), ST (*S*.Tm infected), VD (VD3 and *S*.Tm infected), PP1 or PP5 (1% or 5% propionate and *S*.Tm infected), VD + PP1 (combination of VD3 and 1%propionate plus *S*.Tm infected), VD + PP5 (combination of VD3 and 5% propionate plus *S*.Tm infected), VD + PP5 + AHRi (combination of VD3 and 5% propionate plus *S*.Tm infected and AhR inhibitor). Diarrhea situation scores (**a**) and loss of body weight (**b**) of mice were recoded daily. Segments of cecum were harvested, fixed in formaldehyde, and stained with hematoxylin and eosin. Representative histological images (×20 and ×50 magnification) of cecum from the different experimental groups were shown in (**c**) and the analyzed pathological scores for colitis in (**d**). The data shown are means ± SEM (*n* = 7 mice/group). (* *p* < 0.05, ** *p* < 0.01).

We recently observed [10] the synergistic effects of postbiotic butyrate and VD3 on AMPs and anti-inflammatory responses in *Salmonella* colitis [10]. Propionic acid has a greater distribution in the small intestine than butyric acid. So, it is of interest to investigate the same benefits of PP in the therapy of *Salmonella* colitis. Accordingly, we demonstrated the same benefit of PP on the VD3-mediated reduced severity of *Salmonella* colitis as butyrate did. To confirm and generalize the involvement of AhR in the synergistic effects of PP on the VD3-mediated reduced severity of *Salmonella* colitis, we investigated the cecal pathology of *Salmonella*-infected WT mice after treatment of VD3 and/or PP along with intraperitoneal injection of AhR inhibitor. Consistent with our previous study [12] in the histopathological analysis of H&E-stained cecal sections, we observed obvious pathological colitis in the *Salmonella*-infected WT mice group in Figure 1. In contrast, we observed that combining high-dose PP and VD3 significantly reduced the severity of *Salmonella* colitis in C57BL/6 mice, including the situation of diarrhea, loss of body weight, and pathologic scores; although low-dose PP had a beneficial effect, it was not significant. Furthermore, inhibition of AhR counteracted the synergistic benefit of combined treatment. It suggests that AhR is involved in the synergistic effects of PP on the VD3-mediated reduced severity of *Salmonella* colitis.

### 3.2. The Involvement of AhR in the Synergistic Effects of VD3 and PP on the Local Inflammatory Responses and Antimicrobial Peptide in the Cecum of Salmonella-Colitis Mice

**Figure 2:**
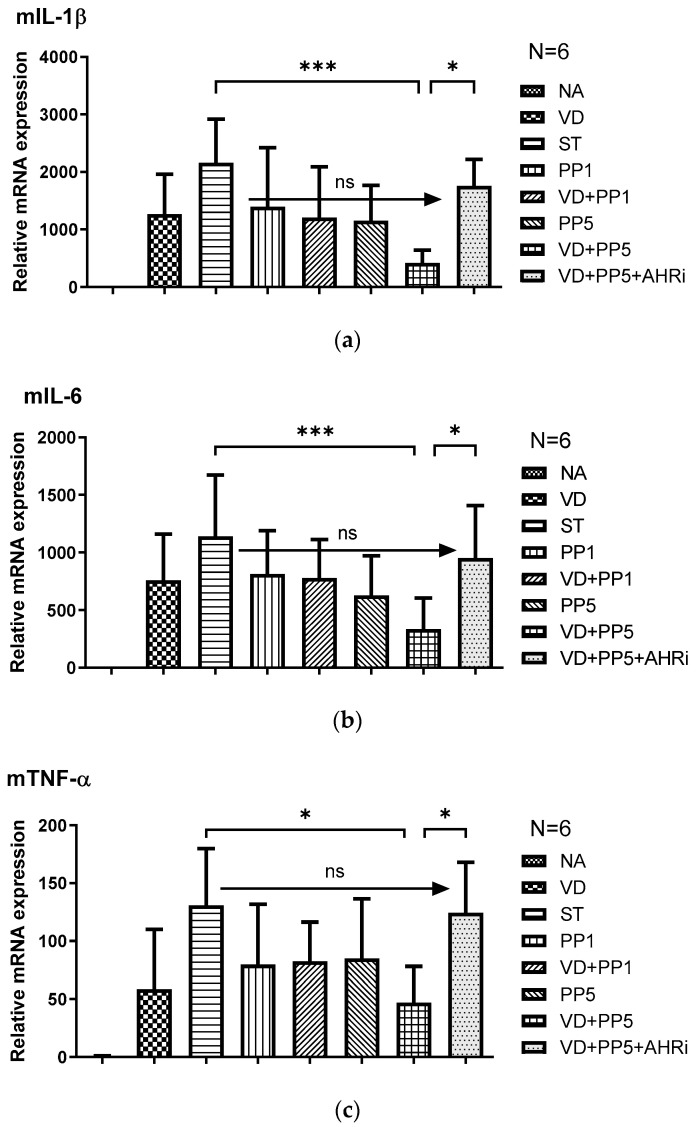

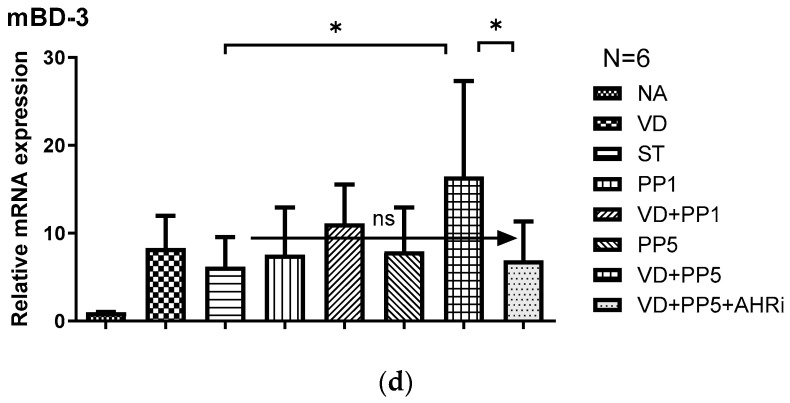
The involvement of AhR in the synergistic effects of PP on the VD3-regulated cecal proinflammatory cytokines and AMP in *Salmonella* colitis mice. Mice were treated or infected as in Materials and Methods and divided into the following groups: Control (NA), ST (*S*.Tm infected), VD (VD3 and *S*.Tm infected), PP1 or PP5 (1% or 5% propionate and *S*.Tm infected), VD + PP1 (combination of VD3 and 1%propionate plus *S*.Tm infected), VD + PP5 (combination of VD3 and 5% propionate plus *S*.Tm infected), VD + PP5 + AHRi (combination of VD3 and 5% propionate plus *S*.Tm infected and AhR inhibitor). Total RNA was extracted from the cecal tissues. IL-1β (**a**), IL-6 (**b**), TNF-α (**c**), and mBD-3 (**d**) mRNA expressions were analyzed using quantitative RT-PCR. Values are measured as fold increase compared to the level of control mice. The data shown are means ± the SEM (*n* = 6 mice/group). (* *p* < 0.05, *** *p* < 0.001, ns: not significant).

Based on our last study [10] that demonstrated the combined effects of postbiotics on VD3-orchestrated anti-inflammatory and anti-bacterial responses in *Salmonella* colitis and the above finding, we proceeded to investigate the involvement of AhR in the combined effects of both supplements on the pro-inflammatory cytokines and AMP expression in *Salmonella* colitis. The gene expression of mIL-1β, mTNF-α, mIL-6, and mBD-3 was quantified using real-time PCR in the cecal tissue of *Salmonella*-infected WT mice after the treatment of VD3 or PP, along with intraperitoneal injection of AhR inhibitor. Cecal gene expression of mIL-1β, mTNF-α, mIL-6, and mBD-3 (Figure 2) was induced in *Salmonella colitis* mice. The pro-inflammatory cytokines were synergistically suppressed after the combined treatment of VD3 and PP, whereas the antimicrobial peptide mBD-3 was synergistically enhanced. Furthermore, inhibition of AhR counteracted the effects on mRNA expressions by treatment, either alone with VD3 or PP, or in combination. It suggests that AhR is involved in the synergistic effects of combined VD3 and PP on the local inflammatory responses and antimicrobial peptide in the cecum of *Salmonella*-infected mice.

Altogether, it suggests AhR is involved in the synergistic effects of PP on the VD3-mediated attenuating severity of *Salmonella* colitis by enhancing anti-inflammatory and antibacterial responses.

### 3.3. Combination of PP Exerted VD3-Mediated Reduction of Bacterial Translocation in Salmonella Colitis Mice

**Figure 3:**
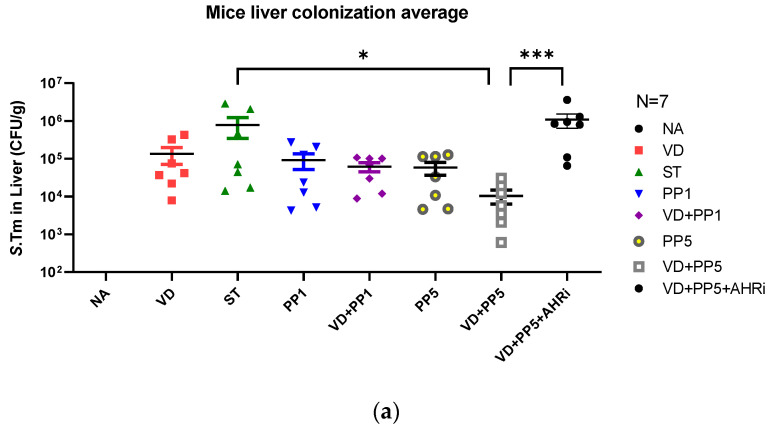

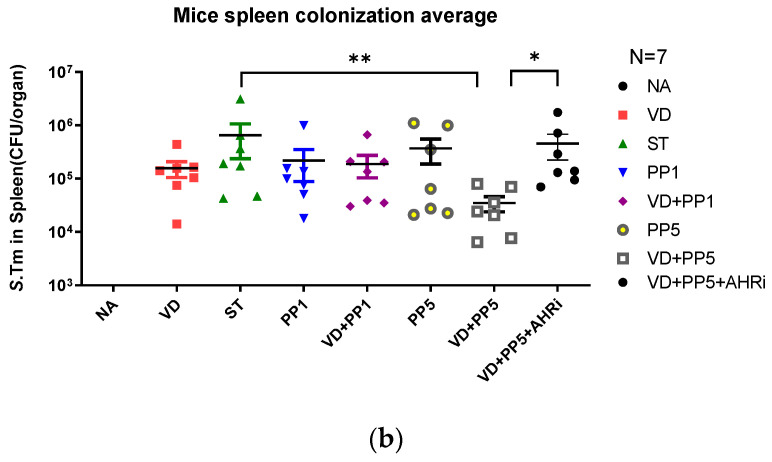
The involvement of AhR in the combined effects of PP and VD3 on attenuating systemic bacterial translocation of *Salmonella colitis* mice. Six–eight-week-old female C57BL/6 mice (Charles River, USA) were obtained from the animal facility of the Center for Cellular and Biomolecular Research, Kaohsiung, Taiwan. Mice were treated or infected as in Materials and Methods and divided into the following groups: Control (NA), ST (*S*.Tm infected), VD (VD3 and *S*.Tm infected), PP1 or PP5 (1% or 5% propionate and *S*.Tm infected), VD + PP1 (combination of VD3 and 1%propionate plus *S*.Tm infected), VD + PP5 (combination of VD3 and 5% propionate plus *S*.Tm infected), VD + PP5 + AHRi (combination of VD3 and 5% propionate plus *S*.Tm infected and AhR inhibitor). Numbers of bacteria were counted from liver (**a**) and spleen (**b**) homogenates of different groups, as shown in Materials and Methods. The data shown are represented as the means ± the SEM of the bacterial load in the liver and spleen (*n* = 7). (* *p* < 0.05, ** *p*< 0.01, *** *p* < 0.001).

Previously, Khailova et al. [14] and our group [12] reported that VD3 can reduce systemic bacterial translocation and mortality in experimental sepsis in mice and *Salmonella* colitis mice. To determine the impact of combined PP treatment on VD3-reduced bacterial invasion, liver and spleen tissues from VD3 and/or PP-treated *Salmonella* colitis mice were obtained, homogenized, and plated on LB plates to determine the CFU. We observed that combined PP treatment exerted a VD3-mediated reduction in bacterial loads in the liver or spleen of *Salmonella* colitis mice (Figure 3).

We observed that the combination of PP and VD3 reduced bacterial colonization (CFU/g tissue) in the liver and spleen, compared to SL1344 infection only, while AhR inhibitor counteracted the combined effects.

### 3.4. Combination of PP and VD3 Exerted Synergistic Effect on VDR and AhR mRNA Expression in Cecal Mucosa of Mice with Salmonella Colitis

Based on our previous observation [15] that VD3 enhanced VDR protein and mRNA expressions in *Salmonella*-infected intestinal epithelial cells and the interaction between VDR and AhR in monocytes/macrophages [16], we studied the effects of combining PP and VD3 on VDR and AhR mRNA expression in cecal tissue in mice with *Salmonella* colitis. By the same animal model, quantitative RT-PCR was used to analyze the gene expression of VDR and AhR in the cecal tissue of infected WT mice in the absence or presence of VD3 or PP, along with intraperitoneal injection of AhR inhibitor.

**Figure 4:**
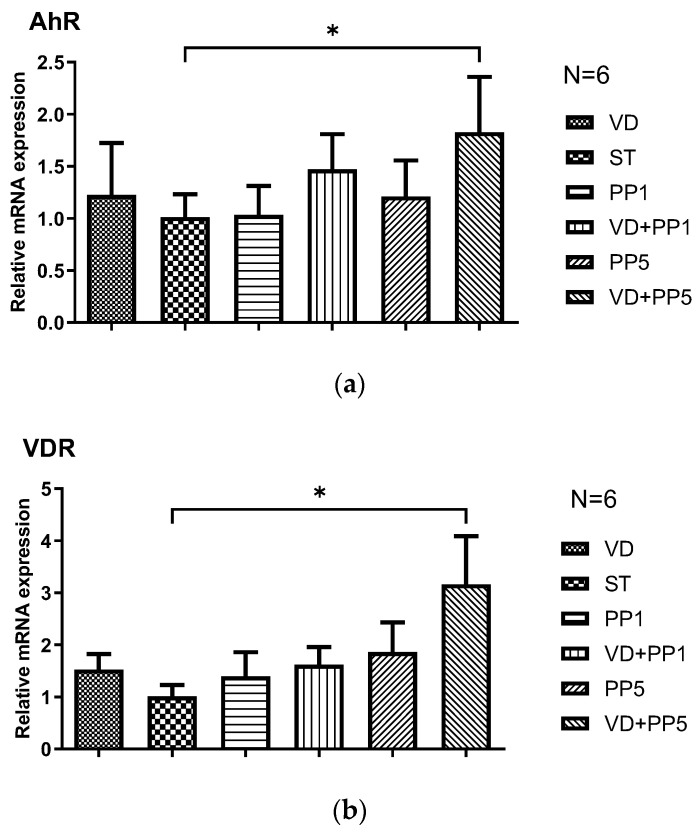
The synergistic effects of PP and VD3 on VDR and AhR mRNA expression in cecal mucosa of mice with *Salmonella* colitis. Mice were treated or infected as in Materials and Methods and divided into the following groups as above: Control, ST, VD, PP1 or PP5, VD + PP1, VD + PP5. Total RNA was extracted from the cecal tissues and quantitative RT-PCR was used to analyze the gene expression of AhR (**a**) and VDR (**b**) in the cecal tissue of infected and/or treated WT mice. Values are measured as fold increase compared to the level of control mice. The data shown are means ± SEM (*n* = 6 mice/group). * *p* < 0.05.

As shown in Figure 4, *Salmonella* infection induced VDR and AhR mRNA expression in the cecal tissue of mice and, the combination of PP and VD3 synergistically enhanced both mRNA expression. It suggests VDR and AhR were both involved in the synergistic effects of PP and VD3.

### 3.5. Combination of PP and VD3 Exerted Synergistic Effect on VDR and AhR Proteins Expression in Cecal Mucosa of Mice with Salmonella Colitis

In order to determine if AhR or VDR protein is involved in the synergistic effects of the treatment combination in *Salmonella* colitis mice, the protein expression of AhR or VDR was analyzed using immunohistochemistry staining on the cecal tissue of *Salmonella* colitis mice in the absence or presence of PP or VD3.

**Figure 5:**
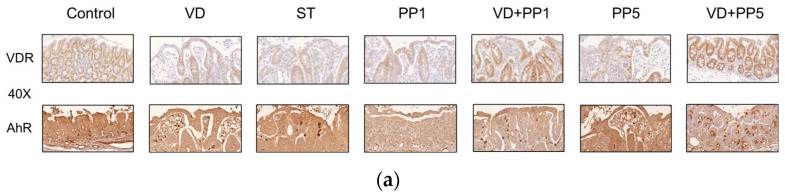

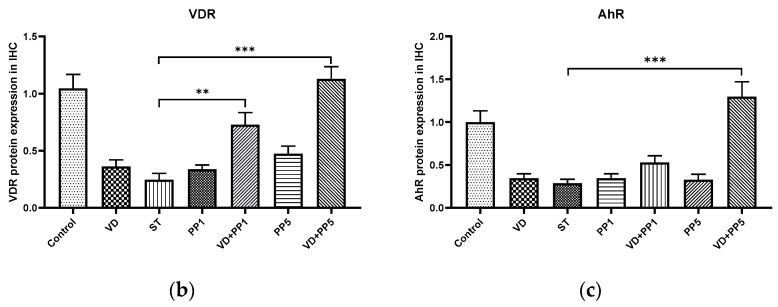
Combination of PP and VD3 synergistically activates the cecal AhR and VDR proteins expression following *Salmonella* colitis in mice. (**a**) AhR and VDR proteins expression in control, ST, VD, PP1, VD + PP1, PP5, and VD + PP5 groups detected by immunohistochemistry staining (original magnification, ×400; scale bar, 25 µm; *n* = 3). The levels of (**b**) VDR and (**c**) AhR immunohistochemistry staining were analyzed and calculated by ImageJ. (** *p*< 0.01, *** *p* < 0.001).

As shown in Figure 5, AhR and VDR proteins were present at significantly increased levels in cecal mucosa in *Salmonella* colitis through the treatment of PP and VD3, compared with the *Salmonella* infection only.

## 4. Discussion

We observed combination of high-dose PP exerted synergistic benefits on VD3-mediated reduced loss of body weight, diarrhea situation scores, and severity of *Salmonella* colitis in C57BL/6 mice along with reduced cecal pro-inflammatory mIL-1beta, mIL-6, mTNF-α mRNAs expression, whereas enhanced antimicrobial mhBD-3 mRNA, compared to single treatment. In contrast, intraperitoneal injection of AhR inhibitor counteracted the synergistic effects of VD3 and postbiotics. It suggests the critical role of AhR in the synergistic benefits of VD3 and postbiotics on *Salmonella* colitis.

Following ligand binding, AhR is translocated to the nucleus, where it forms a heterodimer with the AhR nuclear translocator, thereby inducing AhR-dependent gene expression. Propionate, a potential ligand for AhR, activates the AhR pathway and AhR-dependent genes in human intestinal epithelial cell lines [17], independent of the well-known mechanisms of gene regulation including G-protein coupled receptors GPR41, GPR43, GPR109a, monocarboxylate transporter MCT-1 and inhibition of histone deacetylases (HDAC). Butyrate, as well as phenyl derivatives of butyrate, increased β-defensin 2 and β-defensin 3 expression in a porcine-derived colon cell line [18] and stimulated the expression of β-defensin 2 and β-defensin 3 in the colon and ileum of pigs, which ultimately led to protection against severe infection with *E. coli* [19]. Oral administration of sodium propionate could ameliorate dextran sulfate sodium (DSS)-induced colitis in mice mainly by reducing cecal inflammation, including IL-1β, IL-6, and TNF-α mRNA expression, and oxidative stress [20], while inducing antimicrobial peptides [21], regenerating islet-derived protein type 3 [Reg3] lectins. Clinically, the anti-inflammatory properties of propionate were shown to decrease the severity of colitis in Crohn’s disease (CD)-associated adherent-invasive *Escherichia coli* (AIEC)-infected mice [22]. Propionate levels have been reported to be markedly decreased in patients with CD compared with healthy individuals [23]. Therefore, our data also provide insights into new preventive or curative treatments for CD.

Oral administration of the β-naphthoflavone (an AhR agonist) decreased the severity of colitis and the production of pro-inflammatory cytokines, such as TNF-α, IL-6, and IL-1β in DSS-induced colitis [24]. Treatment of 2,3,7,8-tetrachlorodibenzo-p-dioxin (TCDD), a synthetic AhR ligand, also attenuated the severity of DSS-induced colitis through epigenetic regulation of immunity [25]. Activation of AhR triggered by specific ligands increases regulatory mechanisms such as IL-10, IL-22, prostaglandin E2, and Foxp3 and results in decreased inflammatory responses, such as IFN-γ, IL-6, IL-12, TNF-α, IL-7, and IL-17, along with the production of antimicrobial peptides and epithelial repair, leading to reduced microbial translocation in the gut [26]. AhR^−/−^ mice are hypersensitive to LPS-induced septic shock compared with wild-type (WT) controls, suggesting that AhR negatively regulates LPS-induced serum levels of inflammatory cytokines, including IL-1β, IL-6, and TNF-α, toward Gram-negative bacteria [27,28,29,30]. Rather, the same response is similarly observed for Gram-positive intestinal pathogens, like *Listeria monocytogenes*. AhR deficiency in mice revealed increased production of pro-inflammatory cytokines (e.g., IL-6 and TNF-α) by macrophages after infection, resulting in higher susceptibility to *L. monocytogenes* [31], with higher morbidity and mortality rates [32]. Likewise, administration of FICZ, an AhR agonist, protects against *L. monocytogenes* infection [33]. On the other hand, active vitamin D3 (VD3) decreases cecal inflammatory responses and attenuates the severity of *Salmonella* colitis [12], along with butyrate, to synergistically induce anti-inflammatory and antimicrobial responses via VDR [10]. Furthermore, increased production of inflammatory cytokines to LPS, such as TNF-α and IL-1β, contributed to the failure of intestine barrier integrity in vitamin D deficient and VDR KO mice [34], leading to bacterial translocation, endotoxemia, and mortality. It suggests the combined effects of AhR and VDR ligands on suppressing inflammatory responses and the severity of *Salmonella* colitis.

SCFAs can significantly induce basal and ligand-induced *AhR* mRNA and protein expression in mouse colonocytes and Caco-2 cells, and also enhances the recruitment of the AhR to the target gene promotors [35]. Therefore, SCFAs act together with AhR ligands to increase the activation of this receptor in gut epithelial cells, and to potentiate its anti-inflammatory role. Administration of butyrate also increases intestinal VDR expression and suppresses inflammation in a colitis model [36]. Tributyrin, a prodrug of natural butyrate, induced overexpression of VDR [37] and increased binding of VD3 to its receptor, contributing to the synergistic effect of tributyrin and active VD3 in Caco-2 cells. Propionate promoted VDR expression in the intestine via the activity of the yes-associated protein (YAP), both in vitro and in vivo [38]. Additionally, VD3 increased VDR expression and AhR activation and repressed NF-κB phosphorylation. Furthermore, AhR was identified as a receptor for 1,25(OH)_2_ D3 to some extent, as predicted by molecular modeling [39]. VD3 inhibits LPS-induced IL-6 overexpression in human oral epithelial cells through AhR/NF-κB signaling, which may provide an explanation for the anti-inflammatory effect [40]. Very interestingly, transcriptional attenuation triggered by butyrate or vitamin D3 was reported in colonic carcinoma cells [41]. Accordingly, we demonstrated that the AhR inhibitor counteracts the synergistic effect of propionate and VD3, including inflammatory responses and antimicrobial peptide expression.

Although the direct interaction between AhR and VDR has not been detected, activation of VDR enhanced the gene expression and activity induced by AhR ligands in human monocyte/macrophage cells [16]. Cooperative regulation of the expression of cytochrome P450 enzymes by AhR and VDR was reported [42]. AhR is a molecular target of calcitriol in human T cells [43]. Ligand-activated AhR increased 1,25(OH)_2_D3-dependent recruitment of VDR to the target gene promoter to stimulate vitamin D3 signaling in macrophages [42]. On the other hand, suppression of AhR may be mediated by direct binding of VDR in the *ahr* gene [16]. Indeed, at least 10 potential binding sites of VDR in the *ahr* gene have been identified based on the consensus binding motif [44]. Together, these data implicate that the interaction between vitamin D and AhR ligand may be a critical factor that determines the overall outcomes of immunity.

## 5. Limitations

Although we provide a novel concept that the interaction between AhR and VDR may influence the innate immunity and severity of *Salmonella* colitis, the story will be clearer by using VDR-knockout (KO) and AhR-KO mice, which are not available in our country. Besides, we still have some experiments that should be added to our project, such as the ex vivo study of isolating innate immune cells from the intestinal tissues of colitis mice. Unfortunately, our research grant could only support our team for one-year experiments. In order to study much more about the immune interaction between AhR and VDR, we will continue to apply for the budget subsidy from the Ministry of Science and Technology. We hope the most powerful biotherapy, even nutrients, to cure *Salmonella* colitis can be discovered and a better solution can be found to defend against the disease.

## 6. Conclusions

We observed combination of postbiotics and VD3 synergistically reduced the severity of *Salmonella* colitis and body weight loss in C57BL/6 mice by reducing cecal inflammatory mIL-6, mTNF-α, and mIL-1beta mRNA expressions, but enhancing the antimicrobial peptide mhBD-3 mRNA, compared to single treatment. Inhibition of AhR counteracted the synergistic effects of the combined treatment, either on the severity of colitis or mRNA expressions in mice with *Salmonella* colitis. It suggests AhR is involved in the synergistic benefits of combined postbiotics and VD3 in enhancing antibacterial and anti-inflammatory responses as well as attenuating the severity of *Salmonella* colitis. These findings will not only explore how postbiotics and VD3 can strengthen the human body’s innate immunity against invasion of *Salmonella* infection, but also the critical role of AhR on the combined effects of postbiotics and VD3. Therapies aimed at enhancing AhR activity and optimizing an effective immune response represent exciting avenues of discovery and potential therapeutics for critically ill patients in the future. Globally, it will inevitably make a great contribution to *Salmonella* infection control, and the same theory could be applied to various intracellular pathogens, as well as the therapeutic strategy can be extended to the study of other infections.

## Data Availability

The data presented in this study are available on request from the corresponding author.

## Supplementary Materials

The following supporting information can be downloaded at: https://www.mdpi.com/article/10.3390/biomedicines11010195/s1, Figure S1: Experimental groups and protocol.
