# Supplementary material for: The Critical Role of Acyl Hydrocarbon Receptor on the Combined Benefits of Postbiotic Propionate on Active Vitamin D3-Orchestrated Innate Immunity in Salmonella Colitis
Source: Biomedicines. 2023 Jan 12;11(1):195. doi: 10.3390/biomedicines11010195 (PMC9855671; doi:10.3390/biomedicines11010195)
Supplement: Supplementary file 1 [file biomedicines-11-00195-s001.zip › biomedicines-2046890-supplementary.pptx]

## Slide 1
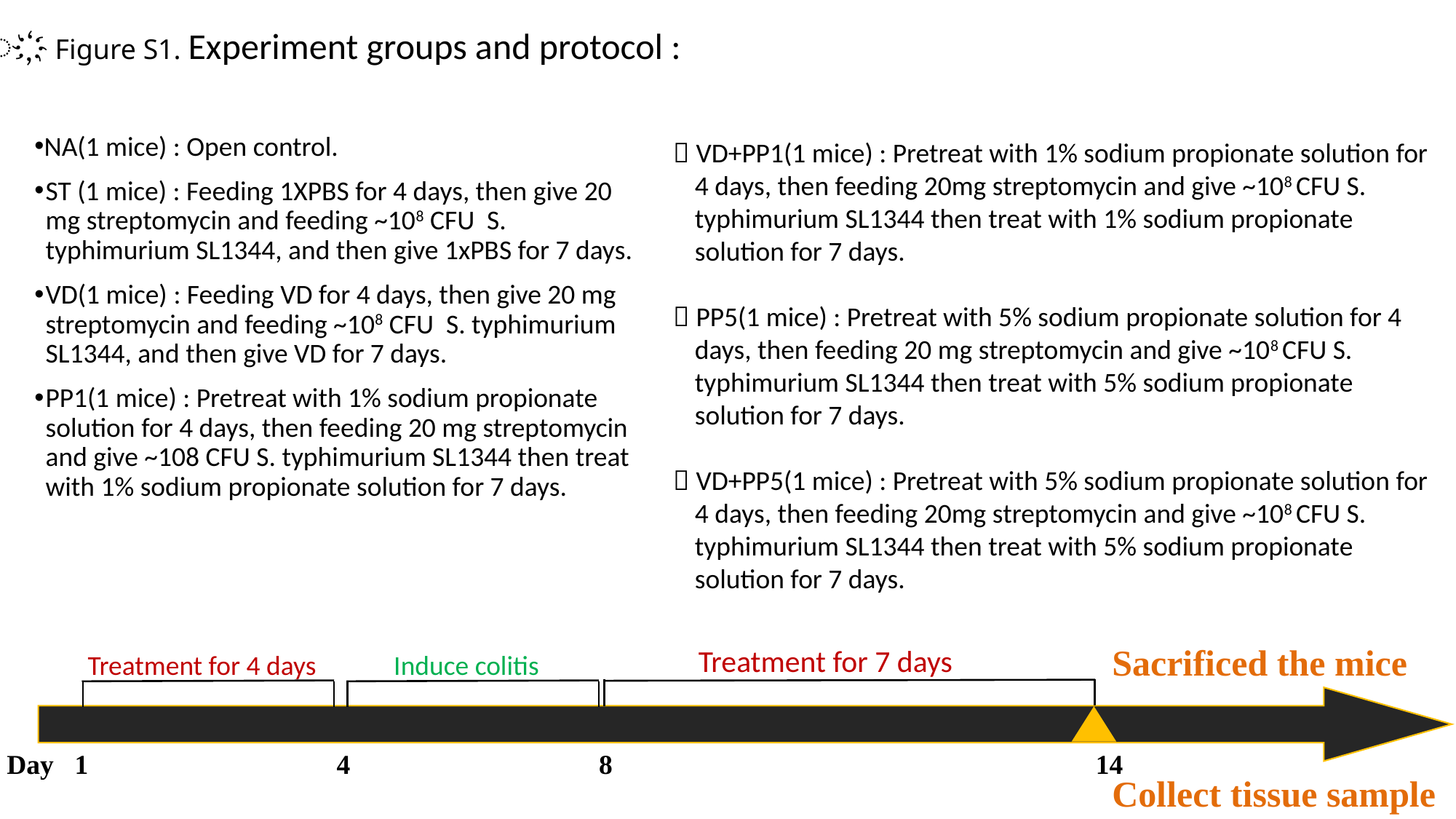

҉ Figure S1. Experiment groups and protocol :
NA(1 mice) : Open control.
ST (1 mice) : Feeding 1XPBS for 4 days, then give 20 mg streptomycin and feeding ~108 CFU S. typhimurium SL1344, and then give 1xPBS for 7 days.
VD(1 mice) : Feeding VD for 4 days, then give 20 mg streptomycin and feeding ~108 CFU S. typhimurium SL1344, and then give VD for 7 days.
PP1(1 mice) : Pretreat with 1% sodium propionate solution for 4 days, then feeding 20 mg streptomycin and give ~108 CFU S. typhimurium SL1344 then treat with 1% sodium propionate solution for 7 days.
．VD+PP1(1 mice) : Pretreat with 1% sodium propionate solution for 4 days, then feeding 20mg streptomycin and give ~108 CFU S. typhimurium SL1344 then treat with 1% sodium propionate solution for 7 days.
．PP5(1 mice) : Pretreat with 5% sodium propionate solution for 4 days, then feeding 20 mg streptomycin and give ~108 CFU S. typhimurium SL1344 then treat with 5% sodium propionate solution for 7 days.
．VD+PP5(1 mice) : Pretreat with 5% sodium propionate solution for 4 days, then feeding 20mg streptomycin and give ~108 CFU S. typhimurium SL1344 then treat with 5% sodium propionate solution for 7 days.
Sacrificed the mice
Collect tissue sample
Treatment for 7 days
Induce colitis
Treatment for 4 days
Day 1 4 8 14
